# Acupoint injection treatment for primary osteoporosis: A systematic review and meta-analysis of randomized controlled trials protocol

**DOI:** 10.1097/MD.0000000000016735

**Published:** 2019-08-09

**Authors:** Rui Huang, Shihong Xu, Dingpeng Li, Xingwen Xie

**Affiliations:** a Clinical Medical College of Traditional Chinese Medicine, Gansu University of Chinese Medicine; b Institute of Osteopathy Gansu Institute of Traditional Chinese Medicine; c Department of Bone Oncology, Gansu Provincial Hospital of Traditional Chinese Medicine, Lanzhou, Gansu, China.

**Keywords:** acupuncture, meta-analysis, osteoporosis, protocol, systematic review

## Abstract

**Background:**

Acupoint injection has currently received increasing attention as a treatment for primary osteoporosis (POP). A number of randomized controlled trials (RCTs) have reported that acupoint injection have some advantages in treatment of POP. However, no article has summarized the existing evidence. This study will evaluate the efficacy and safety of acupoint injection as a clinical treatment for POP, so as to provide an important reference for clinical decision-making.

**Methods:**

RCTs of acupoint injection compared with conventional non-acupoint injection for POP were identified in searches of 7 databases from their inception to March 2019. All data were assessed and extracted by 2 authors independently. The risk of bias assessment recommended by the Cochrane Collaboration was used to assess the quality of the selected studies. Review Manager 5.3 (Cochrane Collaboration) was used to conduct meta-analysis for the efficacy and safety of acupoint injection.

**Result:**

The results of this systemic review and meta-analysis will be submitted to a recognized journal for publication.

**Conclusion:**

This systemic review and meta-analysis will evaluate the efficacy and safety of acupoint injection as a clinical treatment for POP. We hope this study can make a definitive conclusion for acupoint injection in the treatment of POP.

**Registration:**

PROSPERO (registration number CRD42019130890).

## Abstract 2

Supplemental Digital Content is available in the text

## 1 Introduction

Primary osteoporosis (POP) is a metabolic bone disease with clinical characteristics of systemic bone pain, spinal deformity, and increased risk of bone fractures. Senile osteoporosis and postmenopausal osteoporosis are the main components of POP. With the increase in the aging population, the morbidity of osteoporosis is increasing, with an estimated 200 million people affected worldwide.^[1]^ In the United States, approximately 50% of people aged over 50 years are at risk for osteoporotic fracture.^[2]^ More than 60% of osteoporosis patients sustain an associated fracture in their lifetime,^[3,4]^ which seriously hinders the treatment of osteoporosis patients and increases the mortality rate.^[5]^ Many osteoporosis patients die within 1 year of a hip fracture.^[6]^ Therefore, effective strategies to reduce the incidence of fracture are urgently required. In recent years, the problem of osteoporosis has received increasing attention worldwide.^[7–9]^

According to the current practice guidelines, the first-line treatment for POP is anti-osteoporosis medication,^[10,11]^ such as bisphosphonates,^[12]^ denosumab,^[13–15]^ teriparatide,^[16,17]^ and salmon calcitonin.^[18]^ These drugs are administered orally, intramuscularly, or intravenously. In some countries, including China, acupoint injection is often used instead of intramuscular injection to obtain a better curative effect and reduce bad translation.^[19,20]^

Acupoint injection is a supplementary replacement therapy, also known as “water needle,” that involves treating diseases by injecting appropriate medication into relevant acupoints, such as Mingmen (DU 34), Zusanli (ST 36), and Sanyinjiao (SP6). The theory of traditional Chinese medicine holds that acupoint injection reinforces liver and kidney function and strengthens muscles and bones. At the same time, modern theoretical research also shows that acupoint injection can stimulate the body's meridian system, generate bioelectric activities, and regulate the functions of the viscera and nervous system, increase the energy state, and strengthen the normal metabolic function of the body.^[21]^ Some clinical trials of acupoint injection therapy for POP have been reported; however, a systematic evaluation of the efficacy of acupoint injection therapy for POP remains to be conducted.

High-quality meta-analysis is increasingly regarded as a reliable source of research evidence.^[22,23]^ Therefore, we conducted this systematic review to evaluate the efficacy and safety of acupoint injection as a clinical treatment for POP. This information will provide an important reference for clinical decision-making.

## 2 Methods

### 2.1 Study registration and ethics

This protocol has been registered at PROSPERO (registration number: CRD42019130890; http://www.crd.york.ac.uk/PROSPERO). Data from individual patients will not be used in this systemic review and no privacy will be involved. So the ethical approval is not necessary.

### 2.2 Selection criteria

#### 2.2.1 Type of study

We will include randomized controlled trials (RCTs) of acupoint injection for POP; the language of the literature will not be limited. Controlled clinical trials, case reports, review articles, conference abstracts, editorials, letters, and expert opinions will be excluded.

#### 2.2.2 Participants

Studies enroll patients diagnosed as POP that complied with international Reference Standards (the operational definition of osteoporosis is based on the T score for bone mineral density [BMD] assessed by dual energy X-ray absorptiometry at the femoral neck or spine and is defined as a value for BMD 2.5 SD or more below the young female adult mean). Characteristics such as age, sex, and ethnicity were not restricted.

#### 2.2.3 Interventions

The experiment group must be applied alone with acupoint injection, the drug types for acupoint injection, the acupuncture points for injection, treatment frequency, and duration of treatment will not be restricted. The control group is treated with intramuscular injection alone; the drugs for injection should be the same as those in experiment group.

#### 2.2.4 Outcomes

The primary outcomes are fracture incidence, BMD, pain measurement. The secondary outcomes are biochemical markers of bone turnover, adverse effect.

### 2.3 Search strategy

We will search 7 databases including PubMed, Web of Science, EMBASE, Cochrane Library, Chinese National Knowledge Infrastructure (CHKD-CNKI), Chinese Biomedical Literature Database (CBM), and WanFang Database (Chinese Medicine Premier). We will search all the databases from their inception to March 2019 by 2 independent authors. The search terms “point injection,” “acupoint injection,” and “osteoporosis,” “osteopenia,” “bone mineral density,” “bone density.” The medical subject heading terms “osteoporosis,” “acupuncture,” “acupuncture points,” or “injection” will be used. We will adjust the search strategies for each database. Details of the strategies used to search international databases are shown in the supplementary materials.

### 2.4 Data extraction

Data will be extracted by 2 authors independently. All differences will be settled by discussion between the 2 researchers. If we cannot reach an agreement, we will consult a third reviewer. Data extract including the basic information of the trial (name of the first author, year of publication), basic research information (patient information, experimental intervention, control intervention), evaluation time, outcomes (BMD, pain measurement, fracture incidence, etc.), and relevant important variables. If information is missing, we contact the authors of the primary studies.

### 2.5 Quality assessment

Two authors independently evaluated risk of bias in the included RCTs using the Cochrane Collaboration's risk of bias tool.^[24]^ This tool assesses the following domains: random sequence generation, allocation concealment, blinding of participants and personnel, blinding of outcome assessment, incomplete outcome data, selective reporting, and other sources of bias. We will rate each domain as low, unclear, or high risk of bias. We classify the overall risk of bias as low if all domains are at low risk of bias, as high if at least 1 domain is at high risk of bias, or as unclear if at least 1 domain is at unclear risk of bias and no domain is at high risk. This rule is specified by Cochrane's tool for assessing risk of bias in RCTs, because any source of bias in a trial is problematic and there is a paucity of empirical research that supports prioritization of 1 domain over the others.^[25]^

### 2.6 Statistical analysis

#### 2.6.1 Meta-analysis

We will perform meta-analysis using Review Manager Software 5.3 (Cochrane Collaboration). Dichotomous variables will be calculated as relative risk with 95% confidence interval (CI), and continuous variables will be calculated as the mean difference or standardized mean difference with 95% CI.

#### 2.6.2 Measures for heterogeneity

The degree of heterogeneity (*I*^2^) of each outcome will be analyzed using the Chi-squared test, with no significance designate by *P* > .05. *I*^2^ < 50% indicates low heterogeneity of the data and the fixed model adopted for a meta-analysis; otherwise the random effects model will be used. If substantial heterogeneity is detected, subgroup or sensitivity analysis is applied to explore the causes of heterogeneity. If the sources of heterogeneity could not be determined, we adopt descriptive analysis.^[26]^

#### 2.6.3 Publication bias

Funnel plot and Egger's test will be applied to evaluate the existence of publication bias.

## 3 Discussion

A number of RCTs has shown the efficacy and safety of acupoint injection for the treatment of POP. While the participants included in the trials were relatively small, in the same time no article has summarized the existing evidence. Due to the small simple, we cannot judge the efficacy and safety of acupoint injection accurately. Therefore, we conduct this systemic review and meta-analysis so as to provide reliable evidence for clinical promotion of acupoint injection for POP. If acupoint injection for POP is really efficacy and safety, this systemic review could give patients and clinicians several recommendations.

This protocol has been registered, we will strictly execute according to the Cochrane Handbook for Systematic Reviews of Interventions and is presented per the Preferred Reporting items for Systematic Reviews and Meta-analyses (PRISMA) guideline. However, there may be several limitations in this review. Firstly, we include only English and Chinese literatures that will be lead to selection bias. Secondly, for most primary studies, the acupuncture points for injection, treatment frequency and duration of treatment are varied, which may cause heterogeneity. Thirdly, our research is based on present research only; the emergence of new research in the future may have an impact on current results, so we will update the study periodically. In conclusion, we hope this study can make a definitive conclusion for acupoint injection in the treatment of POP.

## Author contributions

**Conceptualization:** Rui Huang.

**Data curation:** Shihong Xu.

**Funding acquisition:** Xingwen Xie.

**Methodology:** Dingpeng Li.

**Project administration:** Xingwen Xie.

**Protocol draft:** Rui Huang, Shihong Xu.

**Software:** Shihong Xu.

**Study design:** Rui Huang, Shihong Xu, Dingpeng Li.

**Validation:** Xingwen Xie.

**Writing – original draft:** Rui Huang.

**Writing – review & editing:** Dingpeng Li.

## Supplementary Material

Supplemental Digital Content
