# Supplementary material for: Acupoint injection treatment for primary osteoporosis: A systematic review and meta-analysis of randomized controlled trials protocol
Source: Medicine (Baltimore). 2019 Aug 9;98(32):e16735. doi: 10.1097/MD.0000000000016735 (PMC6709080; doi:10.1097/MD.0000000000016735)
Supplement: Supplemental Digital Content [file medi-98-e16735-s001.docx]

Search Strategies for Pubmed, Embase,web of scienece and Cochrane Library

**pubmed**

#1 osteoporosis[tiab] OR osteopenia[tiab]

#2 piont injection[tiab] OR acupoint injection[tiab]

#3 osteoporosis[mesh]

#4 Acupuncture[mesh] OR Acupuncture Points[mesh] OR Injection[mesh]

#5 ((“Clinical Trials, Phase II as Topic”[Mesh] OR “Clinical Trials, Phase III as Topic”[Mesh] OR “Clinical Trials, Phase IV as Topic”[Mesh] OR “Controlled Clinical Trials as Topic”[Mesh] OR “Randomized Controlled Trials as Topic”[Mesh] OR “Intention to Treat Analysis”[Mesh] OR “Pragmatic Clinical Trials as Topic”[Mesh] OR “Clinical Trials, Phase II”[Publication Type] OR “Clinical Trials, Phase III”[Publication Type] OR “Clinical Trials, Phase IV”[Publication Type] OR “Controlled Clinical Trials”[Publication Type] OR “Randomized Controlled Trials”[Publication Type] OR “Pragmatic Clinical Trials as Topic”[Publication Type] OR “Single-Blind Method”[Mesh] OR “Double-Blind Method”[Mesh])) OR (random*[Title/Abstract] OR blind*[Title/Abstract] OR singleblind*[Title/Abstract] OR doubleblind*[Title/Abstract] OR trebleblind* [Title/Abstract] OR tripleblind*[Title/Abstract])

#6 #1 or #3

#7 #2 or #4

#8 #5 and #6 and #7

**Embase**

#1 osteoporosis :ab,ti OR osteopenia:ab,ti

#2 piont injection:ab,ti OR acupoint injection:ab,ti

#3 osteoporosis'/exp

#4 Acupuncture'/exp OR Acupuncture Point'/exp OR Injection'/exp

#5 'multicenter study (topic)'/exp OR 'phase 2 clinical trial (topic)'/exp OR 'phase 3 clinical trial (topic)'/exp OR 'phase 4 clinical trial (topic)'/exp OR 'controlled clinical trial (topic)'/exp OR 'randomized controlled trial (topic)'/exp OR 'single blind procedure'/exp OR 'double blind procedure'/exp

#6 random*:ab,ti OR blind*:ab,ti OR singleblind*:ab,ti OR doubleblind*:ab,ti OR trebleblind*:ab,ti OR tripleblind*:ab,ti

#7 #1 OR #3

#8 #2 OR #4

#9 #5 OR #6

#10 #7 AND #8 AND #9

**Web of Science**

#1 osteoporosis :ti OR osteopenia:ti

#2 piont injection:ti OR acupoint injection:ti

#3 #1 AND #2

**Cochrane Library**

#1 (osteoporosis):ti,ab,kw OR (osteopenia):ti,ab,kw

#2 (piont injection) :ti,ab,kw OR (acupoint injection):ti,ab,kw

#3 MeSH descriptor:[Osteoporosis] explode all trees

#4 MeSH descriptor:[Acupuncture] explode all trees

#5 MeSH descriptor:[Acupuncture Point] explode all trees

#6 MeSH descriptor:[Injection] explode all trees

#7 #1 OR #3

#8 # 2 OR #4 OR #5 OR #6

#9 #7 AND #8
